# Peripheral and Central Impact of Methionine Source and Level on Growth Performance, Circulating Methionine Levels and Metabolism in Broiler Chickens

**DOI:** 10.3390/ani13121961

**Published:** 2023-06-12

**Authors:** Craig W. Maynard, Elizabeth Gilbert, Frances Yan, Mark A. Cline, Sami Dridi

**Affiliations:** 1 Center of Excellence for Poultry Science, University of Arkansas, Fayetteville, AR 72701, USA; 2 Department of Animal and Poultry Sciences, Virginia Polytechnic Institute and State University, Blacksburg, VA 24061, USA; 3 Novus International, Saint Charles, MO 63304, USA; 4 School of Neuroscience, Virginia Polytechnic Institute and State University, Blacksburg, VA 24061, USA

**Keywords:** DL-methionine, HMTBa, ICV injection, diet supplementation, hypothalamus, gene expression

## Abstract

**Simple Summary:**

As it is the first limiting amino acid, the use of DL-methionine in poultry diet formulation is critical for poultry growth, yet its mode of action is not fully defined. In this study, we conducted two peripheral and central feeding trials to determine the effect of DL-methionine and its analog HMTBa on growth performance, plasma metabolites, and the gene expression of feeding-related hypothalamic neuropeptides in broilers raised to market age (35 d). The study shows for the first time that both sources of methionine share some pathways, but each one has a unique pathway.

**Abstract:**

The present study was designed to evaluate the effects of DL-methionine (DL-Met) 2-hydroxy-4-(methylthio) butanoic acid (HMTBa), or S-(5′-Adenosyl)-L-methionine chloride (SAM), using feeding trial and central administration, on live performance, plasma metabolites, and the expression of feeding-related hypothalamic neuropeptides in broilers raised to a market age (35 d). Final average body weight (BW) and feed conversion ratio (FCR) from the feeding trial exceeded the performance measurements published by the primary breeder. At d35, the MTBHa group had better BW and lower feed intake, which resulted in a better FCR than the DL-Met group at 87 TSAA to lysine. At the molecular levels, the expression of hypothalamic neuropeptide (NPY) and monocarboxylate transporter (MCT) 2 did not differ between all treated groups; however, the mRNA abundances of hypothalamic MCT1 and orexin (ORX) were significantly upregulated in DL-Met- treated groups compared to the control. The ICV administration of SAM significantly reduced feed intake at all tested periods (from 30 to 180 min post injection) compared to the aCSF-treated group (control). The central administration of HMTBa increased feed intake, which reached a significant level only 60 min post administration, compared to the control group. ICV administration of DL-Met slightly increased feed intake compared to the control group, but the difference was not statistically discernable. Quantitative real-time PCR analysis showed that the hypothalamic expression of NPY, cocaine- and amphetamine-regulated transcript, MCT1, and MCT2 was significantly upregulated in the ICV-HMTBa group compared to the aCSF birds. The hypothalamic expression of the mechanistic target of rapamycin (mTOR), AMP-activated protein kinase (AMPKα1), D-amino acid oxidase, and hydroxyacid oxidase was significantly upregulated in DL-Met compared to the control group. The mRNA abundances of ORX were significantly increased in the hypothalamus of both DL-Met and HMTBa groups compared to the aCSF birds; however, mTOR gene expression was significantly downregulated in the SAM compared to the control group. Taken together, these data show, for the first time, that DL-Met and HMTBa have a common downstream (ORX) pathway, but also a differential central pathway, typically NPY-MCT for HMTBa and mTOR-AMPK for methionine.

## 1. Introduction

Methionine represents the first limiting amino acid in poultry diets and was the first feed-grade amino acid added to poultry feed in order to reduce soybean meal usage and satisfy amino acid requirements [1]. Methionine use in poultry diets dates back to the early 1950s and it is the only amino acid that can be supplied as both of its optical isomers (i.e., DL methionine), allowing for the use of chemically synthesized methionine, although it is commonly supplied as a hydroxy analogue (i.e., 2-hydroxy-4-(methylthio)butanoic acid (HMTBa)). Both forms of supplemental methionine have been shown to be effective in supporting broiler growth, but different responses for the two forms have been reported when dietary levels deviate from broiler requirement estimates [2].

In addition to methionine’s position as the first limiting amino acid in poultry feeds, it has also been identified as the most toxic amino acid, resulting in the greatest depressions in growth among the essential amino acids when offered in excess of requirement estimates [3,4]. While experimental excesses of methionine used to observe its toxic effects were found at levels far exceeding those typically observed in broiler diets, methionine represents a nutritional paradox due to its essentiality and the need to closely monitor its inclusion levels in order to prevent negative effects on broiler performance. Therefore, the present feeding studies were conducted to determine the effects of a moderate deficiency and moderate excess of dietary methionine on growth performance, plasma levels, and target gene expression using its most prevalent supplementary forms. Additionally, in order to define the central mode of methionine and its hydroxy analog’s action, a second study was conducted using the intracerebroventricular (ICV) administration of methionine, HMTBa, S-(5′-Adenosyl)-L-methionine chloride (SAM), or artificial cerebrospinal fluid (aCSF) as a placebo. 

## 2. Materials and Methods

### 2.1. Ethic Statement

All procedures utilized in the present study were approved by the Institutional Animal Care and Use Committee of the University of Arkansas (Fayetteville, AR, USA; Protocol number 21050). 

### 2.2. Bird Husbandry

A total of 1260 male Cobb 500 broiler chicks were obtained from a commercial hatchery (Cobb Vantress, Siloam Springs, AR, USA), where they received Marek’s vaccinations at day (d) of hatch and transported to the University of Arkansas Broiler Research Farm. Upon arrival, chicks were placed into 60 pens at 21 birds per pen and with a density of 0.09 m^2^ per bird. Each pen was equipped with a hanging feeder, section of continuous nipple drinker line (5 nipples per pen), and built-up, top-dressed litter composed of pine shavings. Starter diets were offered as crumbles from d 0 to 18, and grower diets were offered as pellets from d 19 to 35. Feed and water were provided ad libitum throughout the experiment. Water flow rates were set at 21 mL per minute and increased by 7 mL per minute every week. Initial temperatures were set at 32.2 °C and gradually reduced to 18.3 °C at the conclusion of the experiment. Lighting schedules were set at 24L:0D for d 0, 23L:1D from d 1 to 6, and 18L:6D from d 7 to 35. Light intensities were verified at bird level using a light meter (LT300, Extech Instruments, Waltham, MA, USA) and water flow rates were verified using the equipment and methods established by Miles et al. [5].

### 2.3. Feeding Experiment

Broiler starter and grower diets were formulated according to breeder recommendations using 2-hydroxy-4-(methylthio) butanoic acid (HMTBa) as the methionine source (Cobb-Vantress, 2018) (Table 1). Supplemental HMTBa was then increased and decreased by 50 percent at the expense of corn starch to create the three levels of HMTBa that were used. Three supplemental levels of DL-methionine (DL-Met) were calculated on an equal molar basis, resulting in a 2 × 3 factorial arrangement of 6 treatments consisting of methionine source by level. The resulting methionine levels were represented by their average ratio of total sulfur amino acids (TSAA) to lysine across the two dietary phases. A common basal diet was mixed in a one-ton vertical screw mixer and aliquoted for the six dietary treatments. Graded amounts of DL-Met, HMTBa, and corn starch were added to each aliquot, and the experimental diets were mixed, pelleted at 65.5 °C, and bagged. Representative samples were collected during bagging and submitted for analysis (Novus International, St. Charles, MO, USA). Diets were analyzed for total amino acids and HMTBa (Novus Methods RM113; RM115; 121210-6, Rev. 4) (Table 2).

Weekly pen weights and feed consumption were recorded. Body weights were determined by dividing pen weight by the number of birds for each pen. Body weight gain for each cumulative period was determined by subtracting initial body weight from period final body weight. Feed conversion ratio, representing g of feed intake to g of body weight gain, was calculated by dividing pen feed intake by the summation of pen body weight gain and recorded mortality weight for each pen. Feed intake was adjusted for mortality using bird days, as outlined by Greenwood et al. [6]. 

### 2.4. Intracerebroventricular Experiment

Five-day post-hatch male Cobb500 chicks were randomly divided into four body-weight (94 ± 2.8 g average BW, *n* = 10 birds/group)-matched groups and assigned to ICV administration with artificial cerebrospinal fluid (aCSF) as vehicle, HMTBa (Alimet, Novus International, Saint Charles, MO, USA, 16 nmol), DL-methionine (DL-Met, Evonik Industries AG, Essen, Germany, 16 nmol), or S-(5′-Adenosyl)-L-methionine chloride (SAM, Cayman Chemical, Ann Arbor, MI, USA, 16 nmol), as previously described [7]. Briefly, chicks were freehand ICV-injected using an adapted method that does not appear to induce physiological stress [8]. The head of the chick was briefly inserted into a restraining device which approximately orients the skull, as described by Kuenzel and Masson [9]. The injection coordinates were 3 mm anterior to the coronal suture, 1 mm lateral from the midline sagittal suture, and 2 mm deep in the external surface of the skull targeting the left lateral ventricle. Anatomical landmarks were determined visually and by palpation. Injection depth was controlled by placing a plastic tubing sheath over the needle. The needle remained at the injection depth in the chick for 5 s post-injection to reduce backflow. Feed intake was recorded at 30, 60, and 180 min after injection. After data collection, the birds were euthanized by cervical dislocation and the injection site was verified by brain section made along the frontal plane. Any chick without dye present in the lateral ventricle system was discarded. Hypothalamus, and plasma from dye-positive chicks were harvested and kept at −80 °C until use. The dissection of chicken hypothalamus was based on the stereotaxic atlas of the brain of the chick [9]. The hypothalamus, defined by the posterior margin of the optic chiasm and the anterior margin of the mammillary bodies to the depth of approximately of 2–4 mm, was dissected.

### 2.5. Measurement of Plasma Methionine and HMTBa

After the final weighing, one bird per pen from the in-feeding trial and all birds from the ICV experiment were used for blood sampling and collection from the jugular vein. Blood samples were then centrifuged (2000× *g*, 10 min, 4 °C), plasma collected, and stored at −80 °C for later analysis. Plasma samples were sent for determination of free methionine and HMTBa (Inotiv Laboratories, West Lafayette, IN, USA with their internal method, which uses UHPLC with a triple quad MS). 

### 2.6. RNA Isolation, Reverse Transcription, and Quantitative Real-Time PCR

Isolation, integrity assessment, and concentration measurement of hypothalamic total RNA was previously described [7,10,11,12]. Total RNA samples were DNase treated, reverse transcribed using qScript cDNA Sythesis Supermix (Quanta Biosciences, Gaithersburg, MD, USA), and amplified by real-time quantitative PCR (Applied Biosystems 7500 Real Time System) with PowerUp SYBR green master mix (Life Technologies, Carlsbad, CA, USA) as previously described [13,14]. The qPCR cycling conditions and melt curve analysis were also previously reported [10,11,12,13,14]. Relative expression of the target genes was determined using the 2^−ΔΔCT^ method [15], with normalization to 18s rRNA as a housekeeping gene. The control group without methionine from the feeding trial and the aCSF group from the ICV experiment were used as calibrators. The specific oligonucleotide sequences for chicken neuropeptide Y (NPY), agouti-related protein (AgRP), cocaine- and amphetamine-regulated transcript (CART), proopiomelanocortin (POMC), orexin (ORX), orexin receptor 1 and 2 (ORXR1/2), adiponectin (Adip), adiponectin receptor 1 and 2 (Adip-R1/2), adenosine monophosphate (AMP)-activated protein kinase alpha 1 and 2 (AMPKα1/2), mechanistic target of rapamycin (mTOR), neurosecretory protein GL (NPGL), and ribosomal 18S as a housekeeping gene were previously published [16,17,18]. The specific oligonucleotide sequences for chicken D-amino acid oxidase (DAO), hydroxyacid oxidase (HAO), and monocarboxylate transporter 1, 2, and 3 (MCT1-3) are summarized in Table 3.

### 2.7. Statistics

For live performance measurements, pen was considered the experimental unit, and treatments were assigned to pens in a randomized complete block design with pen location serving as the blocking factor. All treatments were represented by 10 replicate pens of 21 birds. Data were analyzed by two-way ANOVA using the PROC MIXED procedure of SAS 9.4 (SAS Institute, 2012) to assess the effects of methionine source, TSAA level, and their subsequent interaction. For gene expression and plasma data, bird was considered the experimental unit and data were analyzed by one-way ANOVA and Tukey’s significant difference test was used as post hoc and multiple comparison test. These analyses were carried out using Graph Pad Prism software (version 7.0 for Windows, La Jolla, CA, USA) and differences were considered significant at *p* < 0.05.

## 3. Results

### 3.1. Feeding Trial

Final BW on d 35 averaged 2.868 kg, with an average FCR of 1.423, exceeding the performance measurements published by the primary breeder (Cobb-Vantress). Final mortality averaged 9.29 percent but was unaffected by dietary treatment (Table 4). A significant level effect was observed, mainly between 63 and 75 TSAA, for lysine diets for BW, BWG, FCR (*p* < 0.05), and FI (*p* = 0.057). Although the source effect was not significant, the MTBHa (MHA) group had a numerically better body weight and lower feed intake, which resulted in a better FCR than DL-Met group at 87 TSAA to lysine (Table 4). At this level, HMTBa also had lower mortality (9.52 vs. 10.48%), although the difference was not statistically discernable. 

To gain better insights, data were analyzed by growing phase. Significant interactions between methionine source and TSAA level were observed for d 7 BW and from 0 to 7 d BWG, but means could not be separated using a Tukey’s post-hoc test (Table 5). No other interactions were identified throughout the experiment (Table 4, Table 5, Table 6, Table 7 and Table 8). Feed conversion for the 0–14 d period were highest (*p* < 0.05) for broilers fed the 63 TSAA to lysine diets, lowest (*p* < 0.05) for broilers fed the 75 TSAA to lysine diets, and intermediate (*p* > 0.05) for broilers fed the 87 TSAA to lysine diets. No differences (*p* > 0.05) in live performance were observed for live performance during the 0–21 d period. Differences in FCR were observed during the 0–28 d period, in which birds fed the 63 TSAA to lysine diets had a higher (*p* < 0.05) FCR when compared with those fed the 75 and 87 TSAA to lysine diets. These differences in FCR persisted until the end of the trial at d 35. For the 0–35 d period, d 35 BW and BWG were lowest (*p* < 0.05) for broilers fed the 63 TSAA to lysine diets, highest (*p* < 0.05) for broilers fed the 75 TSAA to lysine diets, and intermediate (*p* > 0.05) for broilers fed the 87 TSAA to lysine diets.

A significant source by level interaction was observed for plasma-free methionine (Table 9). Broilers fed diets containing DL-methionine with an 87 TSAA to lysine level had the highest (*p* < 0.05) plasma methionine, broilers fed diets containing HMTBa with a 63 TSAA to lysine level had the lowest (*p* < 0.05), and broilers fed diets containing DL-methionine with a 75 TSAA to lysine level were intermediate (*p* < 0.05). Broilers fed diets containing HMTBa had higher plasma HMTBa levels compared with those fed diets containing DL-methionine. 

**Figure 1:**
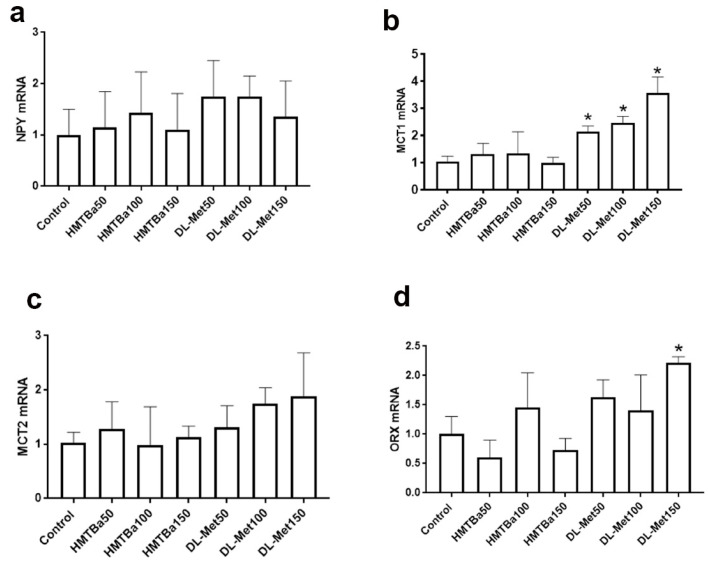
Effects of diets varying in digestible total sulfur amino acids from 0 to 35 d on hypothalamic expression of feeding-related neuropeptides. The control group was fed a basal standard diet with no source of methionine. The mRNA levels of NPY (**a**), MCT1 (**b**), MCT2 (**c**), and ORX (**d**) were determined by qPCR and 2^−ΔΔCT^ [15]. Data are mean ± SEM (*n* = 6–8/group) and * indicates a significant difference compared to the control group at *p* < 0.05. MCT, monocarboxylate transporter; NPY, neuropeptide Y; ORX, orexin.

At molecular levels, the expression of hypothalamic NPY and MCT2 did not differ between all treated groups (Figure 1). However, the mRNA abundances of hypothalamic MCT1 and ORX were significantly upregulated in DL-treated groups compared to the control (Figure 1).

### 3.2. ICV Experiment

**Figure 2:**
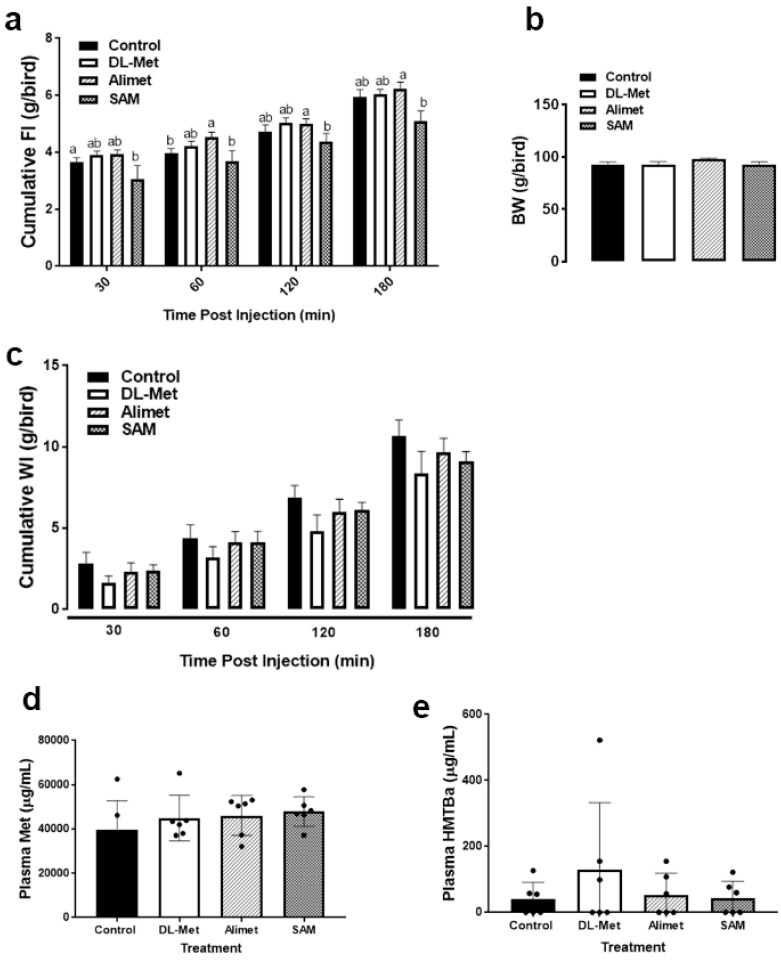
Effects of ICV administration of DL-Met and HMTBa on broiler growth performances and plasma metabolites. (**a**) cumulative FI, (**b**) BW, (**c**) cumulative WI; (**d**) plasma methionine, and (**e**) plasma HMTBa. Data are mean ± SEM (*n* = 10/group) and different letters indicate a significant difference at *p* < 0.05. Alimet, HMTBA (2-hydroxy-4-(methylthio)butanoic acid); DL-Met, DL-methionine; SAM, S-(5′-Adenosyl)-L-methionine chloride.

As shown in Figure 2a, ICV administration of 16 nmols of SAM significantly reduced feed intake compared to the aCSF group at 30 min post injection and compared to Alimet from 60 to 180 min post administration. The central administration of HMTBa (Alimet, 16 nmols) increased feed intake, which reached a significant level only after 60 min post administration, compared to the aCSF group. ICV administration of DL-Met slightly increased feed intake compared to the control group, but the difference was not statistically discernable (Figure 2a). BW and water intake remained unchanged between all studied groups (Figure 2b,c). 

Plasma Met and HMTBa levels increased slightly in the treated (DL-Met, Alimet, and SAM) group compared to the control group, but the difference was not statistically significant (Figure 2d,e). 

**Figure 3:**
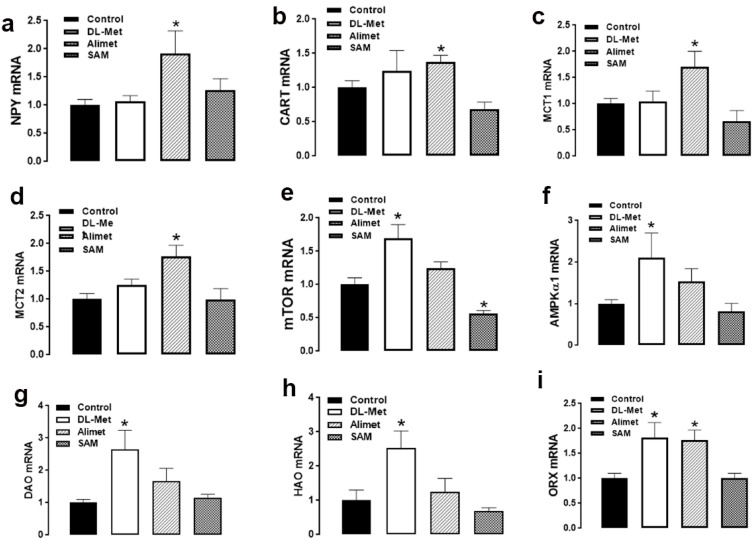
Effects of ICV administration of DL-Met and HMTBa on hypothalamic expression of feeding-related neuropeptides. The gene expression of NPY (**a**), CART (**b**), MCT1 (**c**), MCT2 (**d**), mTOR (**e**), AMPKα1 (**f**), DAO (**g**), HAO (**h**), and ORX (**i**) were determined by qPCR and 2^−ΔΔCT^ [15]. Data are mean ± SEM (*n* = 6–8/group) and * indicates a significant difference compared to the control group at *p* < 0.05. AMPK, adenosine monophosphate (AMP)-activated protein kinase; CART, cocaine- and amphetamine-regulated transcript; DAO, D-amino acid oxidase; HAO, hydroxyacid oxidase; MCT, monocarboxylate transporter; NPY, neuropeptide Y; ORX, orexin.

Quantitative real-time PCR analysis showed that the hypothalamic expression of NPY, CART, MCT1, and MCT2 was significantly upregulated in the ICV-Alimet group compared to the aCSF birds (Figure 3a–d). The hypothalamic expression of mTOR, AMPKα1, DAO, and HAO was significantly upregulated in DL-Met compared to the control group (Figure 3e–h). The mRNA abundances of ORX were significantly increased in the hypothalamus of both DL-Met and HMTBa groups compared to the aCSF birds (Figure 3i). The hypothalamic expression of AgRP, POMC, NPGL, adiponectin system (Adip, AdipR1, AdipR2), orexin receptors (ORXR1, ORXR2), and MCT3 remained unchanged between all studied groups (Table 10). The hypothalamic expression of mTOR was significantly downregulated in the SAM compared to the control group (Figure 3). 

## 4. Discussion

The broiler performance observed in the in-feeding trial largely exceeded the performance objectives published by the primary breeder and likely resulted in the elevated overall mortality observed in the trial. No differences were observed between dietary sources for any performance measurement throughout the trial, indicating that both served as effective sources for dietary methionine. 

Although broilers were fed a diet containing approximately a 16 percent deficiency in TSAA (63 TSAA to lysine diets), broiler performance was not largely impacted. The differences in FCR observed at 14 and 28 d, approximately 1 and 2.5 points, respectively, were driven by the numerical differences in BW gain and feed intake and align with previous reports in the literature examining similar ranges in dietary TSAA levels [19,20]. The separation in d 35 BW and 0–35 d BW gain for broilers fed the diet marginally deficient in methionine can likely be attributed to the increasing methionine requirement relative to lysine as broilers age [21], resulting in 78 g lower BW gain for broilers fed the 63 TSAA to lysine diet when compared with broilers fed the 75 and 87 TSAA to lysine diets. Overall, no toxic effects of excess methionine were observed at the levels used in this study, but these findings are not surprising, as it has been reported that feeding 0.5 percent excess methionine is not harmful to broilers fed corn-soybean meal diets [22].

It has been reported that HMTBa serves as a better methionine source in the event of a methionine overage but DL-methionine is better in the event of methionine deficiency [19]. The results from the plasma analysis reported herein indicated that dietary HMTBa is not immediately converted into methionine and partially remains in the circulating plasma as HMTBa, preventing toxic amounts of methionine from building up in the bloodstream. The improved FCR observed in this study is probably associated with the upregulation of the hypothalamic expression of both MCT1 and ORX. Although further mechanistic studies are needed, mammalian MCT1, which is generally accepted to be a transporter for methionine, and its precursor HMTBa [23], have been found to be expressed in ventral β1 tanycytes, which are in close contact with neurons that express AgRP and NPY [24]. Mammalian ORX has been reported to play pleiotropic physiological functions from the regulation of feed intake and energy homeostasis to the wake/sleep cycle and stress (for review see [25,26]). Furthermore, it has been shown that MCTs interact with the spontaneous firing activity of orexin neurons [27]. However, it is surprising that HMTBa supplementation did not affect the hypothalamic expression of the abovementioned genes, although both HMTBa and DL-Met appear to adequately support bird performance when considering their methionine content on molar equivalence. This could be associated with the intestinal absorption rate [28,29] and/or blood–brain barrier uptake efficacy [30] of both sources, which unfortunately were not measured in our experimental conditions.

To gain better insights into the mode of action of both sources on growth performance (FI, BW, and FCR), we separately ICV-administered DL-Met and HMTBa in the third ventricle, where the centers of hunger and satiety, as well as the key feeding-related neuropeptides, reside. Both sources slightly increased feed intake, with a significant effect observed for HMTBa after 60 min post injection. In contrast, ICV administration of SAM reduced feed intake, which corroborates previous studies, including our own [31,32]. Of particular interest, the upregulation of hypothalamic ORX expression in both HMTBa and DL-Met groups suggest that the two studied sources share a common downstream pathway. Although the direct effect of HMTBa or DL-Met on the central orexin system needs to be delineated, the above result is not surprising because ORX has already been shown to be stimulated by nutritional mixtures of amino acids containing methionine, both in brain slice patch-clamp experiments and in c-Fos expression assays following the central administration of AAs to rodents in vivo [33]. 

In addition, and as well as the common shared ORX pathway, each source of HMTBa and DL-Met seems to have additional unique downstream mediators. In fact, hypothalamic NPY, CART, MCT1, and MCT2 are specific to HMTBa; however, mTOR, AMPKα1, DAO, and HAO are targets for DL-Met. The key role of NPY, CART, and MCTs in the regulation of food intake is well established [24,34,35,36,37,38,39,40,41,42]; however, the concomitant upregulation of the orexigenic NPY and the anorexigenic CART is quite intriguing and suggests that NPY might override the CART signaling in our experimental conditions.

mTOR is well-known as a master nutrient sensor and hypothalamic amino acid sensing has been proposed to involve hypothalamic mTOR [43]. Recently, Zhou and colleagues have demonstrated that methionine regulates mTOR expression via T1R1/T1R3-PLCβ-Ca^2+^-ERK1/2 in C2C12 cells [44]. A more recent study has shown that the taste receptors (TAS1R1-TAS1R3) heterodimer may serve as a sensor of extracellular methionine and that it activates mTOR in bovine mammary epithelial cells [45]. Qi et al. [46], on the other hand, have demonstrated that methionine induces mTOR expression via the degradation of AT-rich interaction domain 1A (ARID1A) in rodent mammary epithelial cells. In yeast, Sutter et al. [47] showed that methionine regulates mTOR expression through the modulation of phosphatase 2A (PP2A) methylation. Similarly, Shen et al. [48] showed that methionine activated the master energy sensor AMPKα in hepatocytes, and mTOR/AMPK (the Yin/Yang of the cellular metabolism) have been extensively reported to be tightly interconnected. Although the directional relationship between AMPK and mTOR is still not known at this timepoint, extensive reports have shown that AMPK inhibits mTOR through at least two mechanisms, namely tuberous sclerosis complex (TSC) and/or the regulatory-associated protein of mTOR (RAPTOR) [49,50,51]. Very recently, and counterintuitively, it has been reported that AMPK directly activates mTOR [52], which is in line with our data. Furthermore, and in support of the present results, Tripodi and co-workers have recently demonstrated that methionine supplementation induced the activation of both the AMPK and mTOR pathways. The upregulation of DAO and HAO expression further supports the abovementioned statement [53] and indicated that methionine is biotransformed in the hypothalamus [54].

Finally, the inhibitory effect of SAM on food intake also seems to be mediated via mTOR, which is not surprising because SAM has been shown to bind SAMTOR, the newly discovered component of the nutrient-sensing pathway upstream of mTOR [55], and SAMTOR has been demonstrated to inhibit mTOR signaling by interacting with GATOR1, the GTPase activating protein (GAP) for RagA/B [55]. As SAM is converted from methionine, one might ask why ICV-administered DL-Met and SAM did not have similar effects. Although in depth-investigations are needed, we speculate that the differential effects observed between DL-Met and SAM was probably associated with the intracellular SAM concentrations and the dissociation constant of the SAM–SAMTOR complex [56]. It is also plausible that SAM can directly serve as a methyl donor and perhaps affect gene expression through methylation, thereby affecting food intake, particularly at later timepoints.

## 5. Conclusions

In conclusion, both HMTBa and DL-methionine appear to adequately support bird performance when considering their methionine content on molar equivalence. Lowered TSAA-to-lysine ratios did not appear to influence performance at younger ages but as the bird ages, increased TSAA levels appear to be necessary to maintain growth performance measurements. At molecular levels, the central effects of DL-Met and HMTBa seem to be mediated via common (ORX) and differential (NPY, MCTs, mTOR, AMPK) pathways.

## Figures and Tables

**Table 1:** Composition of starter (0 to 18 d) and grower (19 to 35 d) diets fed to broilers from 0 to 35 days ^1^.

Ingredient, % As-Is	Starter	Grower
Corn	57.24	63.17
Soybean meal	34.73	28.74
Meat and bone meal	3.00	3.00
Poultry fat	2.38	2.55
L-lysine HCl	0.11	0.13
L-threonine	0.01	0.02
Salt	0.32	0.31
Dicalcium phosphate	0.28	0.17
Choline chloride, 60%	0.05	0.06
Limestone	0.97	0.95
Corn starch	0.11–0.37	0.12–0.35
HMTBa	0.00–0.50	0.00–0.45
DL-Met	0.00–0.43	0.00–0.38
Phytase	0.01	0.01
Sodium bicarbonate	0.17	0.19
Mineral premix ^2^	0.10	0.10
Vitamin premix ^3^	0.10	0.10
Calculated composition, % unless noted otherwise ^4^		
AME, kcal/kg	3031	3108
CP	23.08	20.70
Ca	0.92	0.87
Available P	0.46	0.43
Na	0.20	0.20
Digestible lysine	1.20	1.07
Digestible threonine	0.79	0.72

^1^ Diets contained variable methionine supplementation from either HMTBa or DL methionine. Shown diets represent the HMTBa 75 diets (T3) but HMTBa content varied from 0.00 to 0.50% and DL methionine varied from 0.00 to 0.43%. Corn starch was used to bring diets up to 100%. ^2^ The mineral premix contributed (per kg of diet): manganese, 96.0 mg; zinc, 57.6 mg; copper, 2.7 mg; iodide, 1.9 mg; selenium, 0.2 mg. ^3^ The vitamin premix contributed (per kg of diet): vitamin A, 9259 IU; vitamin D_3_, 6614 ICU; vitamin E, 66 IU; niacin, 46 mg; d-pantothenic acid, 12 mg; riboflavin, 8 mg; pyridoxine, 3 mg; thiamine, 2 mg; menadione, 2 mg; folic acid, 1 mg; biotin, 0.1 mg; vitamin B_12_, 0.02 mg. ^4^ Basal diet found to contain total amino acid levels of (% as-is): 1.19, Lys; 0.36, Met; 0.69, TSAA; 0.80, Thr; 0.90, Val; 0.83, Ile; 1.69, Leu for the starter diet and 1.12, Lys; 0.33, Met; 0.64, TSAA; 0.74, Thr; 0.85, Val; 0.76, Ile; 1.56, Leu for the grower period.

**Table 2:** Analyzed (A) and calculated (C) HMTBa and DL methionine content for starter and grower diets fed from 0 to 35 d post-hatch.

		HMTBa 63	HMTBa 75	HMTBa 87	DL-Met 63	DL-Met 75	DL-Met 87
Starter							
Total Met	A	0.37	0.35	0.36	0.50	0.65	0.81
Total TSAA	A	0.71	0.67	0.69	0.85	0.99	1.13
HMTBa ^1^	C	0.148	0.296	0.445	0.000	0.000	0.000
HMTBa	A	0.144	0.294	0.383	0.029	0.001	0.001
DL-Met	C	0.000	0.000	0.000	0.143	0.285	0.428
DL-Met	A	0.010	0.010	0.010	0.100	0.230	0.380
Grower							
Total Met	A	0.34	0.33	0.32	0.43	0.54	0.72
Total TSAA	A	0.66	0.64	0.63	0.73	0.85	1.02
HMTBa ^1^	C	0.131	0.263	0.394	0.000	0.000	0.000
HMTBa	A	0.103	0.206	0.428	0.001	0.001	0.001
DL-Met	C	0.000	0.000	0.000	0.127	0.253	0.380
DL-Met	A	0.000	0.000	0.000	0.110	0.190	0.290

^1^ Calculated based on molar concentration of HMTBa in MHA (Novus International, St. Charles, MO, USA).

**Table 3:** Oligonucleotide real-time qPCR primers.

Gene	Accession Number ^a^	Primer Sequence (5’ → 3’)	Orientation	Product Size (bp)
*MCT1*	NM_001006323	GCATCTTTGGGAGTTCTGTTGAT	Forward	69
		CCTATCGTGCTCCAACAAACC	Reverse	
*MCT2*	NM_001199604	TCTGGGTTTGGCATTCAACTT	Forward	66
		CGCTTCTTATAGAAATACTTGCCAATC	Reverse	
*MCT3*	NM_205140	GGGTCCGCCCTCATGTG	Forward	69
		TGTCGAGCCATTGAAGAGCAT	Reverse	
*DAO*	XM_040684675	ATGGAACACCATGGGATAGAAGA	Forward	63
		CCTTACCATTGCCACAGAAATG	Reverse	
*HAO*	NM_001199442	CTAGCTCTGGGCGCCAAA	Forward	58
		AAACCAAGCCCCAGATGAGA	Reverse	

^a^ Accession number refers to Genbank (NCBI). DAO, D-amino acid oxidase; HAO, hydroxyacid oxidase; MCT, monocarboxylate transporter.

**Table 4:** Live performance of male Cobb 500 broilers fed diets varying in digestible total sulfur amino acids from 0 to 35 d.

Treatment ^1^		BW, d 35	BWG	FI	FCR	Mortality
Source	Level	(kg)	(kg)	(kg)	(g:g)	(%)
Interactive effects of methionine level and source (*n* = 10)						
HMTBa	63	2.804	2.763	3.937	1.455	6.67
HMTBa	75	2.942	2.902	3.972	1.402	8.10
HMTBa	87	2.936	2.895	3.875	1.383	9.52
DL-Met	63	2.921	2.880	3.949	1.414	10.00
DL-Met	75	2.973	2.933	3.936	1.376	10.95
DL-Met	87	2.911	2.870	3.897	1.399	10.48
SEM		0.0335	0.0335	0.0299	0.0155	2.081
Main effect of methionine level (*n* = 20)						
	63	2.862 ^b^	2.822 ^b^	3.943	1.434 ^a^	8.33
	75	2.958 ^a^	2.917 ^a^	3.954	1.389 ^b^	9.52
	87	2.924 ^a,b^	2.883 ^a,b^	3.886	1.391 ^b^	10.00
SEM		0.0237	0.0237	0.0211	0.0110	1.472
Main effect of methionine source (*n* = 60)						
HMTBa		2.894	2.853	3.928	1.413	8.10
DL-Met		2.935	2.895	3.927	1.396	10.48
SEM		0.0194	0.0193	0.0173	0.0090	1.202
*p*-value						
Interaction		0.114	0.110	0.582	0.187	0.833
Source		0.137	0.136	0.981	0.188	0.167
Level		0.021	0.020	0.057	0.007	0.713

^a,b^ Means separated using a Tukey’s test when found to be significantly different (*p* ≤ 0.05). ^1^ Treatments identified by methionine source and the average methionine to lysine ratio (Met/Lys) fed over the entire growout period.

**Table 5:** Live performance of male Cobb 500 broilers fed diets varying in digestible total sulfur amino acids from 0 to 7 d.

Treatment ^1^		BW, d 7	BWG	FI	FCR
Source	Level	(kg)	(kg)	(kg)	(g:g)
Interactive effects of methionine level and source (*n* = 10)					
HMTBa	63	0.170 ^a^	0.129 ^a^	0.142	1.106
HMTBa	75	0.177 ^a^	0.137 ^a^	0.146	1.077
HMTBa	87	0.175 ^a^	0.135 ^a^	0.143	1.073
DL-Met	63	0.178 ^a^	0.137 ^a^	0.146	1.072
DL-Met	75	0.176 ^a^	0.136 ^a^	0.143	1.066
DL-Met	87	0.173 ^a^	0.132 ^a^	0.143	1.099
SEM		0.0021	0.0020	0.0022	0.0126
Main effect of methionine level (*n* = 20)					
	63	0.174	0.133	0.144	1.089
	75	0.177	0.136	0.145	1.071
	87	0.174	0.132	0.143	1.072
SEM		0.0015	0.0020	0.0015	0.0089
Main effect of methionine source (*n* = 60)					
HMTBa		0.174	0.133	0.144	1.085
DL-Met		0.176	0.135	0.144	1.079
SEM		0.0012	0.0011	0.0012	0.0072
*p*-value					
Interaction		0.031 ^2^	0.022 ^2^	0.341	0.063
Source		0.356	0.325	0.880	0.546
Level		0.276	0.207	0.780	0.325

^a^ Means separated using a Tukey’s test when found to be significantly different (*p* ≤ 0.05). ^1^ Treatments identified by methionine source and the average methionine to lysine ratio (Met/Lys) fed over the entire growout period. ^2^ Post hoc test unable to separate means.

**Table 6:** Live performance of male Cobb 500 broilers fed diets varying in digestible total sulfur amino acids from 0 to 14 d.

Treatment ^1^		BW, d 14	BWG	FI	FCR
Source	Level	(kg)	(kg)	(kg)	(g:g)
Interactive effects of methionine level and source (*n* = 10)					
HMTBa	63	0.515	0.474	0.546	1.165
HMTBa	75	0.534	0.494	0.558	1.143
HMTBa	87	0.525	0.484	0.552	1.151
DL-Met	63	0.528	0.487	0.553	1.155
DL-Met	75	0.535	0.495	0.555	1.145
DL-Met	87	0.527	0.486	0.554	1.157
SEM		0.0056	0.0056	0.0055	0.0060
Main effect of methionine level (*n* = 20)					
	63	0.521	0.481	0.549	1.160 ^a^
	75	0.535	0.494	0.556	1.144 ^b^
	87	0.526	0.485	0.553	1.154 ^a,b^
SEM		0.0040	0.0040	0.0039	0.0042
Main effect of methionine source (*n* = 60)					
HMTBa		0.525	0.484	0.552	1.153
DL-Met		0.530	0.490	0.554	1.152
SEM		0.0032	0.0032	0.0032	0.0035
*p*-value					
Interaction		0.514	0.497	0.637	0.387
Source		0.253	0.252	0.661	0.924
Level		0.055	0.057	0.425	0.033

^a,b^ Means separated using a Tukey’s test when found to be significantly different (*p* ≤ 0.05). ^1^ Treatments identified by methionine source and the average methionine to lysine ratio (Met/Lys) fed over the entire growout period.

**Table 7:** Live performance of male Cobb 500 broilers fed diets varying in digestible total sulfur amino acids from 0 to 21 d.

Treatment ^1^		BW, d 21	BWG	FI	FCR
Source	Level	(kg)	(kg)	(kg)	(g:g)
Interactive effects of methionine level and source (*n* = 10)					
HMTBa	63	1.124	1.083	1.304	1.222
HMTBa	75	1.142	1.102	1.316	1.208
HMTBa	87	1.140	1.100	1.309	1.210
DL-Met	63	1.148	1.108	1.327	1.219
DL-Met	75	1.172	1.132	1.331	1.205
DL-Met	87	1.137	1.096	1.312	1.216
SEM		0.0134	0.0134	0.0131	0.0061
Main effect of methionine level (*n* = 20)					
	63	1.136	1.096	1.315	1.220
	75	1.157	1.117	1.324	1.207
	87	1.139	1.098	1.310	1.213
SEM		0.0095	0.0094	0.0092	0.0043
Main effect of methionine source (*n* = 60)					
HMTBa		1.136	1.095	1.310	1.213
DL-Met		1.152	1.112	1.323	1.213
SEM		0.0078	0.0077	0.0075	0.0035
*p*-value					
Interaction		0.414	0.394	0.754	0.670
Source		0.135	0.129	0.209	0.979
Level		0.238	0.241	0.580	0.096

^1^ Treatments identified by methionine source and the average methionine to lysine ratio (Met/Lys) fed over the entire growout period.

**Table 8:** Live performance of male Cobb 500 broilers fed diets varying in digestible total sulfur amino acids from 0 to 28 d.

Treatment ^1^		BW, d 28	BWG	FI	FCR
Source	Level	(kg)	(kg)	(kg)	(g:g)
Interactive effects of methionine level and source (*n* = 10)					
HMTBa	63	1.955	1.914	2.493	1.326
HMTBa	75	2.006	1.965	2.486	1.300
HMTBa	87	1.997	1.957	2.467	1.292
DL-Met	63	1.990	1.950	2.514	1.317
DL-Met	75	2.022	1.981	2.511	1.294
DL-Met	87	1.991	1.950	2.465	1.299
SEM		0.0191	0.0191	0.0221	0.0051
Main effect of methionine level (*n* = 20)					
	63	1.972	1.932	2.503	1.321 ^a^
	75	2.014	1.973	2.498	1.297 ^b^
	87	1.994	1.954	2.466	1.295 ^b^
SEM		0.0135	0.0135	0.0157	0.0036
Main effect of methionine source (*n* = 60)					
HMTBa		1.986	1.945	2.482	1.306
DL-Met		2.001	1.961	2.496	1.303
SEM		0.0110	0.0110	0.0128	0.0029
*p*-value					
Interaction		0.557	0.543	0.813	0.206
Source		0.334	0.332	0.424	0.569
Level		0.108	0.108	0.200	<0.001

^a,b^ Means separated using a Tukey’s test when found to be significantly different (*p* ≤ 0.05). ^1^ Treatments identified by methionine source and the average methionine to lysine ratio (Met/Lys) fed over the entire growout period.

**Table 9:** Plasma methionine and HMTBa levels.

Treatment ^1^		Methionine	HMTBa
Source	Level	(mmol/mL)	(mmol/mL)
Interactive effects of methionine level and source (*n* = 10)			
HMTBa	63	64.8 ^d^	1.9
HMTBa	75	103.1 ^c,d^	15.2
HMTBa	87	135.3 ^b,c^	25.8
DL-Met	63	109.1 ^c,d^	0.2
DL-Met	75	171.2 ^b^	0.1
DL-Met	87	246.4 ^a^	0.0
SEM		12.37	5.24
Main effect of methionine level (*n* = 20)			
	63	86.9 ^c^	1.0
	75	137.2 ^b^	7.6
	87	190.8 ^a^	12.9
SEM		8.37	3.55
Main effect of methionine source (*n* = 60)			
HMTBa		101.1	14.3
DL-Met		175.6	0.1
SEM		6.73	2.85
*p*-value			
Interaction		0.023	0.063
Source		<0.001	0.001
Level		<0.001	0.069

^a–d^ Means separated using a Tukey’s test when found to be significantly different (*p* ≤ 0.05). ^1^ Treatments identified by methionine source and the average methionine to lysine ratio (Met/Lys) fed over the entire growout period.

**Table 10:** Effect of ICV administration of DL-Met, Alimet, SAM, or aCSF (control) on hypothalamic gene expression.

Gene ^1^	Control	DL-Met	Alimet	SAM
AgRP	1 ± 0.03	1.30 ± 0.10	1.67 ± 0.40	1.33 ± 0.20
POMC	1 ± 0.04	1.08 ± 0.10	1.51 ± 0.20	1.09 ± 0.09
NPGL	1 ± 0.01	1.22 ± 0.20	1.57 ± 0.30	1.13 ± 0.10
Adip	1 ± 0.01	2.41 ± 0.80	1.99 ± 0.50	2.26 ± 0.40
AdipR1	1 ± 0.02	1.33 ± 0.07	1.19 ± 0.10	0.98 ± 0.06
AdipR2	1 ± 0.01	1.03 ± 0.10	1.09 ± 0.08	0.85 ± 0.06
ORXR1	1 ± 0.01	1.02 ± 0.10	1.43 ± 0.30	0.90 ± 0.10
ORXR2	1 ± 0.01	1.24 ± 0.20	1.97 ± 0.70	0.74 ± 0.09
AMPKα2	1 ± 0.01	1.19 ± 0.30	1.38 ± 0.30	0.40 ± 0.10
MCT3	1 ± 0.01	1.99 ± 0.80	1.79 ± 0.40	1.35 ± 0.30

^1^ Adip, adiponectin; AdipR, adiponectin receptor; AgRP, agouti-related peptide; AMPK, adenosine monophosphate (AMP)-activated protein kinase; MCT, monocarboxylate transporter; NPGL, neurosecretory protein GL; ORXR, orexin receptor; POMC, proopiomelanocortin.

## Data Availability

Raw data were generated at the author’s institutions a nd data supporting the findings of this study are available within the article.
